# Analysis of the Cytotoxicity of Carbon-Based Nanoparticles, Diamond and Graphite, in Human Glioblastoma and Hepatoma Cell Lines

**DOI:** 10.1371/journal.pone.0122579

**Published:** 2015-03-27

**Authors:** Karolina Ewa Zakrzewska, Anna Samluk, Mateusz Wierzbicki, Sławomir Jaworski, Marta Kutwin, Ewa Sawosz, André Chwalibog, Dorota Genowefa Pijanowska, Krzysztof Dariusz Pluta

**Affiliations:** 1 Department of Hybrid Microbiosystem Engineering, Nalecz Institute of Biocybernetics and Biomedical Engineering PAS, Warsaw, Poland; 2 Division of Nanobiotechnology, Faculty of Animal Science, Warsaw University of Life Sciences, Warsaw, Poland; 3 Department of Veterinary Clinical and Animal Sciences, Faculty of Health and Medical Sciences, University of Copenhagen, Copenhagen, Denmark; University of Sassari, ITALY

## Abstract

Nanoparticles have attracted a great deal of attention as carriers for drug delivery to cancer cells. However, reports on their potential cytotoxicity raise questions of their safety and this matter needs attentive consideration. In this paper, for the first time, the cytotoxic effects of two carbon based nanoparticles, diamond and graphite, on glioblastoma and hepatoma cells were compared. First, we confirmed previous results that diamond nanoparticles are practically nontoxic. Second, graphite nanoparticles exhibited a negative impact on glioblastoma, but not on hepatoma cells. The studied carbon nanoparticles could be a potentially useful tool for therapeutics delivery to the brain tissue with minimal side effects on the hepatocytes. Furthermore, we showed the influence of the nanoparticles on the stable, fluorescently labeled tumor cell lines and concluded that the labeled cells are suitable for drug cytotoxicity tests.

## Introduction

The characteristic features of nanoparticles (NPs), namely their small size (at least one dimension that measures 100 nanometers or less), high surface area per mass unit and dominating surface properties, provide potential for their application in biomedicine. Carbon NPs are most often used in applications such as drug delivery, bioengineering, biosensors or bioimaging [1]. Despite the similar composition of various carbon NPs, they have distinct physical and biological properties depending on their structure [2]. Diamond NPs (nanodiamond, ND) are characterized by low toxicity and high biocompatibility to a variety of cell types. ND produces low level of reactive oxygen species (ROS), does not stimulate macrophages to produce inflammatory cytokines and does not affect the morphology of cells at concentrations ranging from 1 to 100 μg/mL [3]. In contrast, the biological activity of graphite NPs (nanographite, NG) is poorly understood. There are only a few published reports on this subject, suggesting that NG is capable of inducing apoptosis and cell death or inflammatory responses in rats [4], or could inhibit angiogenesis [5]. Despite the similarity, in terms of having a crystalline form and nanoscale size, ND and NG have different C-atoms hybridization (sp^3^ and sp^2^, respectively) and, thus, exhibit distinct physical and electrochemical properties. This could explain their differential effects exerted on human cells.

According to the World Health Organization cancers are among the leading causes of death throughout the world, and liver cancer is the second most frequent cause of cancer-related death [6]. Hepatocellular carcinoma (HCC) is a primary malignancy of the liver. HCC cells produce proteins at high levels and, thus, they are characterized by high oxygen and glucose consumption [7]. Prognosis for this type of cancer is very poor, because the survival rate of patients with HCC has not been improved significantly in the last two decades [8,9]. The only effective treatment for HCC is surgery (partial resection or transplantation), but only a small percentage of patients are candidates for this procedure, owing to complications associated with the tumor metastasis. Conventional therapy based on chemo- and radiotherapy is toxic to hepatocytes [10]. Glioblastoma multiforme (GBM) is the most common and most aggressive malignant brain tumor. GBM cells are characterized by low mitochondrial respiration, increased glycolysis for ATP generation and hypoxia preference [11]. They are resistant to the traditional therapy and, additionally, the blood-brain barrier limits the penetration of drugs to the tumor site. New strategies developed for cancer treatment are based on substances causing programmed cell death. However, targeted chemotherapeutic agents also have an impact on healthy cells [12,13]. Owing to the problems caused by the blood-brain barrier and to the difficult access to glioblastoma growing along the vasculature and nerves, studies are focusing on targeted therapy, which should not be toxic to the other cells, especially hepatocytes. One of the most promising methods is the use of NPs as carriers for anti-tumor agents.

The aim of this study was to evaluate the potential toxicity of ND and NG in glioblastoma (U87) and hepatoma (C3A) cells. Fluorescent labeling has been widely used in many biological applications, such as in the detection of cellular components (e.g. mitochondria), visualization of protein-protein interactions or *in vivo* cell tracking. Therefore, for the purpose of these experiments, EGFP (enhanced green fluorescent protein)-expressing U87 and C3A cells generated according to a method described elsewhere [14], were used. The experiments with the stable fluorescent cell lines (U87-EGFP and C3A-EGFP) were performed in order to compare the performance of the nontransduced and transduced cells as preliminary studies for future *in vivo* experiments. EGFP-labeling could potentially be toxic to human cells [15], but our data did not confirm this hypothesis because of the following results: unchanged albumin production and viability of the C3A-EGFP cells [16].

## Materials and Methods

### Ethics statement

The Ministry of Environment of the Republic of Poland has granted our Laboratory the approval for research on human cell lines modified by lentiviral vectors for use in closed systems (Decision No. 30/2011).

### Nanoparticles

**Fig 1:**
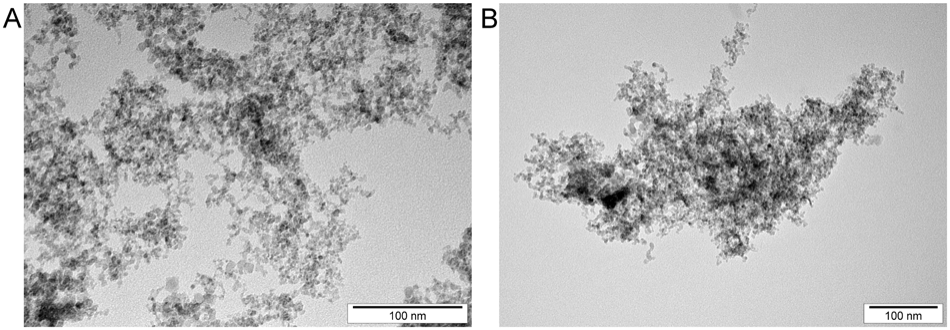
Transmission electron microscopy images of nanoparticles. Images of (**A**) diamond (ND) and (**B**) graphite (NG) nanoparticles. Scale bar: 100 nm.

Carbon–based NPs, ND (explosion synthesized; specific surface area: ~282 m^2^/g; purity: >95%) and NG (explosion synthesized; specific surface area: 540–650 m^2^/g; purity: >93%), were obtained from Sky Spring Nanomaterials Inc. (Huston, USA). ND and NG powders were dispersed in ultrapure water by sonication to prepare 1.0 mg/mL solutions. Afterwards, the solutions were diluted to different concentrations with cell culture medium immediately prior to cell exposure. The dispersion, shape and size of the NPs were examined using a transmission electron microscope (TEM, JEM-2000EX) at an accelerating voltage of 80 kV (JEOL, Tokyo, Japan). Representative TEM images of ND and NG, taken in aqueous solutions at a concentration of 1.0 mg/mL, are presented in Fig 1. As can be seen, NG, but not ND, formed tightly aggregated clusters. The typical diameter of both ND and NG spherical particles ranged from 2–6 nm. The zeta potentials and polydispersity index (PDI) of NPs were measured in colloidal solutions at concentration of 50 μg/mL by the laser dynamic scattering-electrophoretic method, using a Zetasizer Nano ZS, model ZEN3500 (Malvern Instruments, UK). Each sample was measured following stabilization for 120 s at 25°C. The zeta potential measurements were conducted using the Smoluchowski approximation. Observed zeta potentials for ND and NG were −35.6 mV and 31.4 mV, respectively. PDI values for ND and NG were 0.29 and 0.41, respectively.

### Cell cultures

Human glioblastoma U87 (ATCC No. HTB-14) and hepatoma C3A (ATCC No. CRL-10741) cell lines, as well as their fluorescently labeled derivatives, were cultured under standard conditions at 37°C in a humidified atmosphere of 5% CO_2_/95% air in an AutoFlow NU-4750E Water Jacket CO_2_ Incubator (NuAire, Plymouth, MN, USA). U87 cells were cultured in low glucose Dulbecco’s Modified Eagle Medium (DMEM) supplemented with 10% fetal bovine serum (FBS), whereas C3A cells were grown in high glucose DMEM supplemented with 10% FBS and 1% nonessential amino acids.

In order to generate labeled cell lines, both U87 and C3A cells were transduced with lentiviral vectors that enabled the stable expression of the EGFP fluorescent marker. A full description of this genetic modification has been published elsewhere [14]. The resulting fluorescent cell lines (transduced cells): U87-EGFP and C3A-EGFP, displayed strong and uniform fluorescence emissions in a high percentage of cells, that is 90–95% as judged by flow cytometry (fluorescence-activated cell sorting, FACS) using a FACSCanto II instrument (BD, Warsaw, Poland).

### Cell morphology

U87 and U87-EGFP cells were plated in 96-well microplates (1×10^4^ cells per well) and incubated for 18 h. C3A and C3A-EGFP cells were plated in 96-well microplates (3×10^4^ cells per well) and incubated for 24 h. The numbers of cells that allowed optimum growth over the entire incubation period were determined for every cell line in the pilot experiments. All cells were subsequently incubated with ND and NG at concentrations of 20, 50 and 100 μg/mL. Cells cultured in medium without the addition of any NPs were used as a control. Images showing the cell morphology were captured using an inverted fluorescence microscope (Olympus IX71) and analyzed with CellP software (Olympus, Warsaw, Poland) 2 and 24 h after exposure.

### Cell viability

Cell viability (*I*_*CV*_) was assessed using a 3-(4,5-dimethylthiazol-2-yl)-2,5-diphenyltetrazolium bromide reagent (MTT test). U87, U87-EGFP, C3A and C3A-EGFP cells were plated in 96-well microplates (1×10^4^ per well for glioblastoma cells and 3×10^4^ for hepatoma cells) and incubated for 18 or 24 h, respectively. Next, both types of cells were incubated for 2 or 24 h with ND and NG at concentrations of 20, 50 and 100 μg/mL. After removing the medium, the cells were incubated with 50 μL of the MTT solution at 37°C for 3 h. Next, the optical density (OD) of each well was recorded at 570 nm with a microplate reader (Synergy HT) and analyzed using KC-4 software (BioTek, Winooski, VT, USA). The *I*_*CV*_ values were expressed as ratios of the relative optical density for the tested samples (*OD*_*test*_−*OD*_*blank*_) to relative optical density for the control sample (*OD*_*control*_ −*OD*_*blank*_), both relative values were calculated versus the optical density for blank, Equation (1):

$$I_{cv}=\left(OD_{test}-OD_{blank}\right)/(OD_{control}-OD_{blank})$$ (1)

where *OD*_*test*_ is the optical density of cells exposed to ND or NG, *OD*_*control*_ is the optical density of the control sample and *OD*_*blank*_ is the optical density of the wells without glioblastoma or hepatoma cells.

### Cytotoxicity

The cells status, expressed as Cell Index (CI) corresponding to cell number, the morphology and their adherence, was monitored using a real-time xCELLigence RTCA SP cell analyzer (ACEA Biosciences, Inc., San Diego, CA, USA) based on an electronic cell sensor, which measures changes in the electrical properties of cell—growth surface interactions. U87, U87-EGFP, C3A and C3A-EGFP cells were plated in an RTCA SP 96-well microplate (1×10^4^ U87 cells per well and 3×10^4^ C3A cells per well) and left at room temperature for 30 min to allow cell attachment. Then, the plates were transferred into the RTCA SP instrument and incubated for 5 (U87 and U87-EGFP) or 18 h (C3A and C3A-EGFP). The cell numbers that allowed optimum growth over the entire incubation period were determined for every cell line in the pilot experiments. Next, both types of cells were incubated in culture medium containing ND and NG at concentrations of 20, 50 and 100 μg/mL. Cells cultured in a medium without the addition of ND and NG were used as a control. The background CI measured in the medium containing only NPs was at level 0. The CI was monitored for 2 h with a sampling time of 1 min. RTCA software (ACEA) was used for data acquisition and analysis.

### Cell proliferation

Cell proliferation was evaluated using a Cell Proliferation ELISA BrdU kit (Roche Diagnostics GmbH, Germany). U87, U87-EGFP, C3A and C3A-EGFP cells were plated in a 96-well microplate (5×10^3^ U87 cells per well and 3×10^4^ C3A cells per well) and incubated for 24 h. Then, the medium was removed and the cells were incubated with ND and NG at concentrations of 20, 50 and 100 μg/mL. A BrdU reagent was added to each well and incubated for a further 4 h. Further steps were performed according to the Roche Diagnostics protocol. The absorbance was measured at 450 nm using a microplate reader (Infinite M200, Tecan, Durham, NC, USA). Cell proliferation (*I*_*CP*_) was expressed as a ratio of relative optical density for tested samples (*OD*_*test*_−*OD*_*blank*_) to relative optical density for the control sample (*OD*_*control*_−*OD*_*blank*_), both relative values were calculated versus the optical density for the blank, Equation (2):

$$I_{CP}=\left(OD_{test}-OD_{blank}\right)/(OD_{control}-OD_{blank})$$ (2)

### Albumin production

The concentration of human serum albumin secreted by the C3A and C3A-EGFP cells was measured using a sandwich enzyme-linked immunosorbent assay (ELISA) with a quantitation kit (Bethyl Laboratories, Inc., Montgomery, TX, USA) and a microplate reader (Synergy HT, BioTek). C3A and C3A-EGFP cells were plated in 96-well plates (3×10^4^ cells per well) and incubated for 24 h. Then, the cells were further incubated for 24 h with ND and NG at concentrations of 20, 50 and 100 μg/mL. Cells cultured in a medium without the addition of ND and NG were used as a control. The amount of albumin was expressed in nanograms per 1×10^4^ C3A and C3A-EGFP cells.

### Statistical analysis

Data were analyzed using one-way or two-way analysis of variance (ANOVA) with Statgraphics Plus 4.1 (StatPoint Technologies, Warrenton, VA, USA). A *P*-value that was lower than 0.05 was considered to be statistically significant. The differences between the groups were analyzed using Duncan’s multiple range test. The results are presented as the mean values with the standard errors for each variable.

## Results

### Analysis of cell morphology

**Fig 2:**
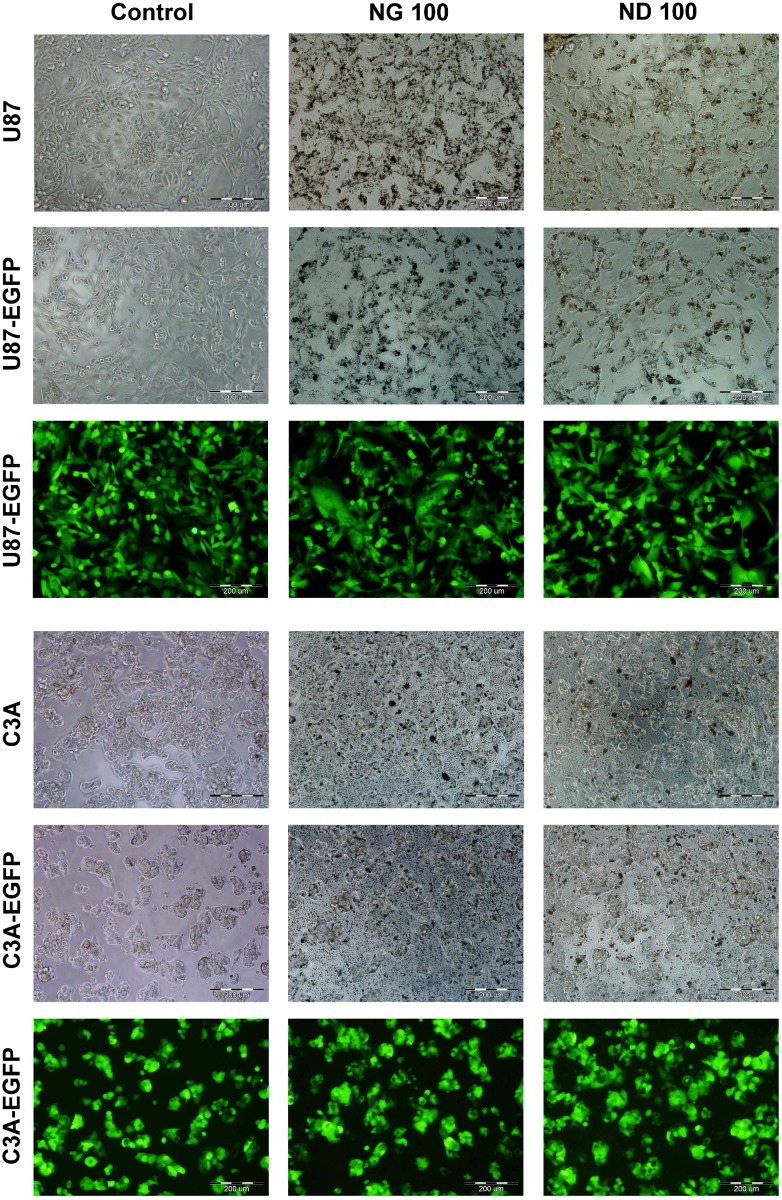
Glioblastoma and hepatoma cells morphology. U87, U87-EGFP, C3A and C3A-EGFP cells morphology after 2 h exposure to medium containing nanoparticles at the highest concentration: graphite 100 μg/mL (NG 100) and diamond 100 μg/mL (ND 100), nontreated cells were used as a control (Control). Analysis of the cells morphology was performed using an inverted fluorescence microscope. Images of the labeled cells, U87-EGFP and C3A-EGFP, were captured using a green fluorescence filter. Scale bar: 200 μm.

The morphology of the tumor cell lines was analyzed using an inverted fluorescence microscope 2 and 24 h after exposure to ND and NG. Regardless of the incubation time, we did not observe any differences in cell morphology compared to the nontreated cells. The NPs appeared on the bright field microphotographs as black dots, aggregated inside the cells or on the cells surface (Fig 2).

### Comparison of cell viability

**Fig 3:**
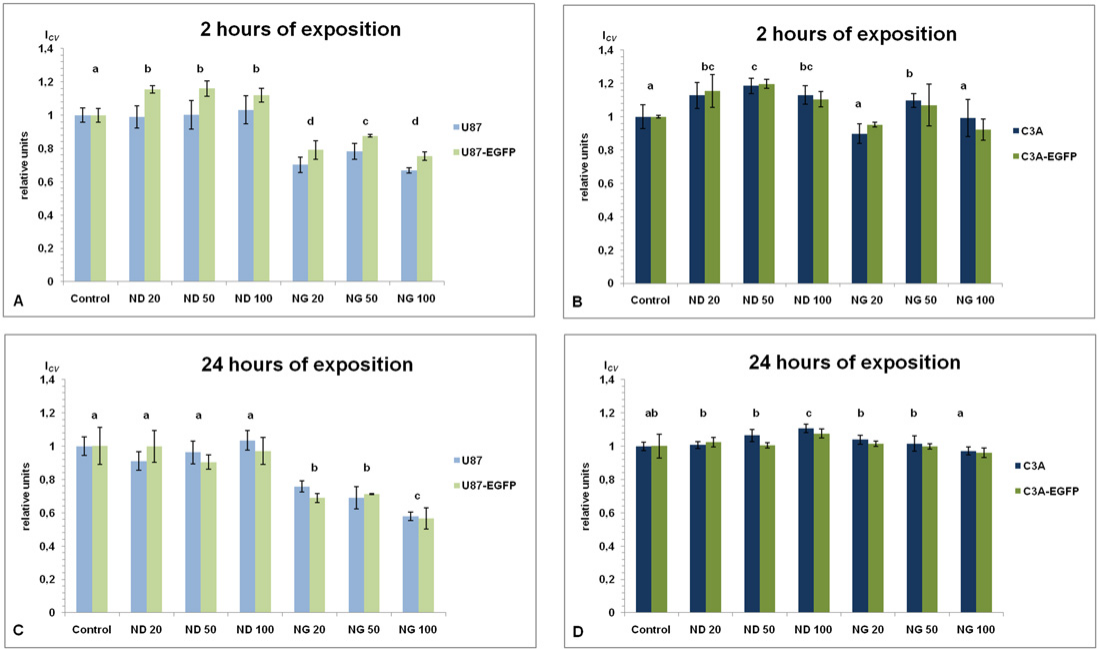
MTT test analysis. U87, U87-EGFP, C3A and C3A-EGFP cells metabolic analysis was performed after 2 and 24 h of exposure to the culture medium containing nanoparticles at different concentrations: diamond 20 μg/mL (ND 20), 50 μg/mL (ND 50) and 100 μg/mL (ND 100), and graphite 20 μg/mL (NG 20), 50 μg/mL (NG 50) and 100 μg/mL (NG 100); nontreated cells were used as a control (Control). Metabolic activity of cells, expressed as *I*_*CV*_, after 2 h of exposure to the culture medium with the above-mentioned different concentrations of diamond and graphite nanoparticles: U87 and U87-EGFP (**A**), and C3A and C3A-EGFP (**B**); and after 24 h of exposure to the culture medium with above-mentioned different concentrations of diamond and graphite nanoparticles: U87 and U87-EGFP (**C**), and C3A and C3A-EGFP (**D**). Data analysis was carried out by two-way ANOVA and the differences between the groups were tested by Duncan’s test. Data were averaged from three replicates (n = 3); *P* < 0.05. No significant interaction (nanoparticles with transduction) was observed.

The viability of the tested tumor cell lines was evaluated using the MTT assay. The glioblastoma cells treated for 2 h with NG at all concentrations showed significantly lowered metabolic activity, by 28.3 ± 5.9% for U87 and 19.3 ± 6.4% for U87-EGFP when compared to the control (Fig 3A). A significantly decreased cell viability, by 32.5 ± 9.1% for U87 and 34.4 ± 7.8% for U87-EGFP, was also noticeable after 24 h of exposure to the NG-containing medium (Fig 3C).

For the hepatoma cell line, we did not observe any significant differences in metabolic activity after either 2 or 24 h of exposure, despite the small decrease by 4.7 ± 1.5% in C3A-EGFP after 2 h exposure to NG at a concentration of 20 μg/mL (Fig 3B; Fig 3D). Analysis of the effect of different ND concentrations on the studied tumor cell lines, U87 and C3A, did not show any negative impact on the cell viability after exposure for 2 or 24 h (Fig 3).

**Fig 4:**
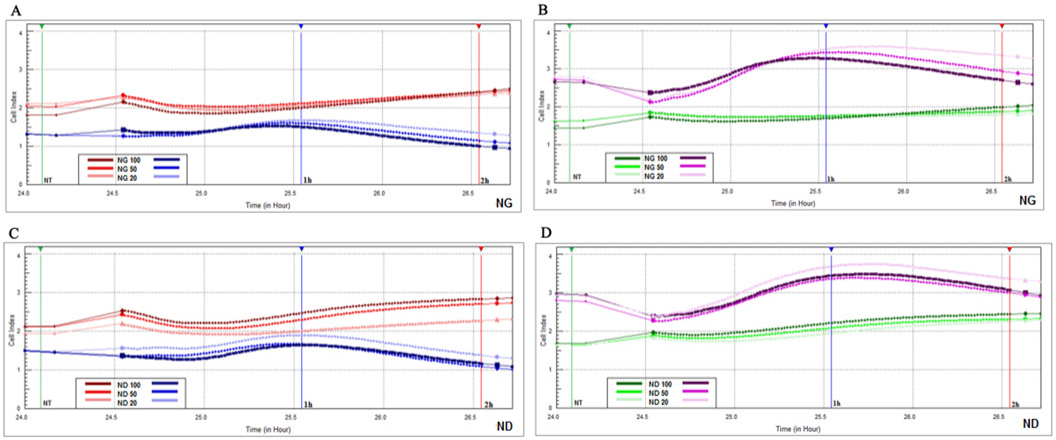
Glioblastoma and hepatoma cells status (CI). U87, U87-EGFP, C3A and C3A-EGFP CI monitored by a real-time cell analyzer (RTCA) after 1 and 2 h of exposure to the culture medium containing graphite and diamond nanoparticles at different concentrations: diamond 20 μg/mL (ND 20), 50 μg/mL (ND 50) and 100 μg/mL (ND 100), and graphite 20 μg/mL (NG 20), 50 μg/mL (NG 50) and 100 μg/mL (NG 100); nontreated cells were used as a control (NT). (**A**) U87 (blue lines) and C3A cells (red lines) treated with graphite (NG) after 1 h (vertical blue marker line) and 2 h (vertical red marker line) and control (vertical green marker line); (**B**) U87-EGFP cells (violet lines) and C3A-EGFP cells (green lines) treated with graphite after 1 h (vertical blue marker line) and 2 h (vertical red marker line) and control (vertical green marker line); (**C**) U87 (blue lines) and C3A cells (red lines) treated with diamond (ND) after 1 h (vertical blue marker line) and 2 h (vertical red marker line) and control (vertical green marker line); (**D**) U87-EGFP cells (violet lines) and C3A-EGFP cells (green lines) treated with diamond (ND) after 1 h (vertical blue marker line) and 2 h (vertical red marker line) and control (vertical green marker line). Data analysis was carried out by two-way ANOVA and the differences between the groups were tested by Duncan’s test. Data were averaged from three replicates (n = 3); *P*< 0.05. No significant interaction (nanoparticles with transduction) was observed.

**Fig 5:**
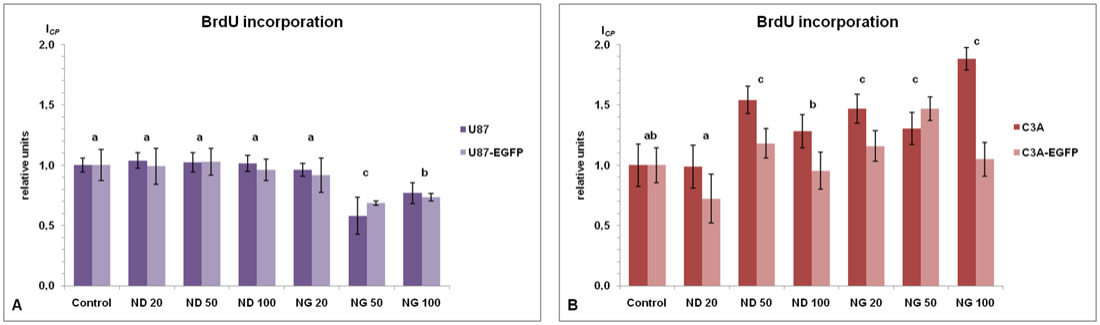
BrdU incorporation test. U87, U87-EGFP, C3A and C3A-EGFP cells proliferation (expressed as *I*_*CP*_) analysis was performed 24 h after treatment with the culture medium containing nanoparticles at different concentrations: diamond 20 μg/mL (ND 20), 50 μg/mL (ND 50) and 100 μg/mL (ND 100), and graphite 20 μg/mL (NG 20), 50 μg/mL (NG 50) and 100 μg/mL (NG 100); nontreated cells were used as a control (Control). (**A**) U87 and U87-EGFP cells and (**B**) C3A and C3A-EGFP cells after 24 h of exposure to the medium with different concentrations of diamond and graphite nanoparticles. Data analysis was carried out by two-way ANOVA and the differences between the groups were tested by Duncan’s test. Data were averaged from three replicates (n = 3); *P*< 0.05. We observed significant interactions (nanoparticles with transduction) in glioblastoma cells (*P* = 0.0001).

**Fig 6:**
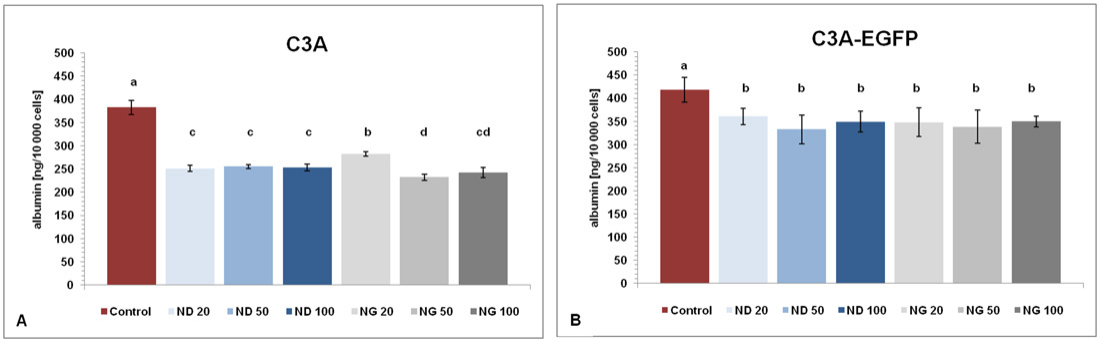
Human serum albumin concentration in culture medium. Albumin secreted by the C3A and C3A-EGFP cells treated for 24 h with the culture medium containing nanoparticles at different concentrations measured by ELISA test: diamond 20 μg/mL (ND 20), 50 μg/mL (ND 50) and 100 μg/mL (ND 100), and graphite 20 μg/mL (NG 20), 50 μg/mL (NG 50) and 100 μg/mL (NG 100). Albumin secretion by cells after 24 h exposure to the medium with different concentrations of diamond and graphite nanoparticles: (**A**) C3A cells and (**B**) C3A-EGFP cells. Nontreated cells were used as a control (Control). Data were analyzed using a two-way ANOVA with the Duncan’s test. Data were averaged from three replicates (n = 3); *P* < 0.05. No significant interaction (nanoparticles with transduction) was observed.

To exclude a possible negative impact of fluorescent protein expression on the metabolic activity in the transduced cells, one-way ANOVA using Duncan’s test, was performed for the control groups (nontreated cells) at two time points (2 and 24 h). We did not observe any statistically significant differences between the genetically modified and unlabeled cells (*P*>0.994 for glioblastoma cells and *P*>0.985 for hepatoma cells). To determine the potential impact of fluorescent protein production on the cells, a series of experiments was performed. In general, no significant alterations in cell morphology or physiology were observed (compare Figs 2–6).

### Cytotoxicity

#### Cytotoxicity of the graphite nanoparticles

Among the four groups of cells tested, only unlabeled U87 showed a significant CI decrease (by 22.3%) after 2 h of exposure to a medium containing NG at a concentration of 100 μg/mL (Fig 4A; dark blue line). Interestingly, when the hepatoma cells were incubated in the culture medium with the highest concentration of NG we observed a CI increase for both C3A and C3A-EGFP cells after 1 h (8 and 12%, respectively) and 2 h of exposure (24 and 28%, respectively) with respect to the CI values measured before the addition of NG (Fig 4A; Fig 4B; dark red and dark green lines, respectively).

#### Cytotoxicity of the diamond nanoparticles

Data from our experiments showed that ND had a broader spectrum of activity, yet it had lower cytotoxicity in the U87 cells compared to NG. After 1 h of exposure, we observed a statistically significant, approximate 10% decrease in CI for U87 at 50 μg/mL and 100 μg/mL, and a 7.3% decrease at the highest concentration for U87-EGFP cells (Fig 4C; Fig 4D; blue/dark blue and dark violet lines, respectively). When the same cells were treated with medium containing ND for 2 h, a cytotoxic effect was only observed in nontransduced U87 cells (Fig 4C; blue and dark blue lines).

The impact of ND on the CI of the hepatoma cells was similar to that observed for NG. The CI increased for C3A and C3A-EGFP after 1 h of exposure to all culture medium containing ND at different concentrations (Fig 4C; Fig 4D; all red and all green lines, respectively). Moreover, when C3A and C3A-EGFP cells were treated with ND for 2 h, a significant growth in the CI was observed at all concentrations. On the other hand, the largest increase, by about 24% for C3A and 32% for C3A-EGFP, was noticed at the highest concentration of ND (Fig 4C; Fig 4D; dark red and dark green lines, respectively).

### Cell proliferation

The proliferative potential of the studied tumor cell lines was evaluated using the BrdU incorporation test. Glioblastoma cells, both unlabeled and EGFP-positive, treated for 24 h with the culture medium containing NG at concentrations 50 and 100 μg/mL showed significantly retarded cell proliferation, expressed as *I*_*CP*_, when compared to the control, by about 38% on average (Fig 5A). For the hepatoma cell lines, we observed a significant increase in *I*_*CP*_ after 24 h of exposure to ND at a concentration of 50 μg/mL and to medium containing NG at all concentrations, when compared to the control, by about 31% on average (Fig 5B). Based on a one-way ANOVA with the Duncan’s test, no statistically significant differences between transduced and nontransduced cells, of both U87 and C3A, were observed.

### Comparison of human serum albumin secretion

To test the liver cell function specifically, the amounts of albumin secreted by C3A and C3A-EGFP cells treated for 24 h with the culture medium containing ND or NG at different concentrations (20, 50 and 100 μg/mL) were determined by the ELISA test. Data showed that, when compared to the control (nontreated cells), the presence of NPs in culture medium significantly lowered the synthesis of the protein in both types of the human hepatoma cells (n = 3; *P*<0.05) (Fig 6A; Fig 6B). The cells treated with ND secreted, on average, about 34 and 17% less albumin for C3A and C3A-EGFP, respectively. In the case in which hepatoma cells were exposed to NG, albumin secretion was lower by the same values in C3A and C3A-EGFP cells when compared to the control.

## Discussion

In this study, for the first time, the potential toxicity of ND and NG in nontransduced and EGFP-expressing glioblastoma and hepatoma cells was evaluated. Earlier reports have shown a constant improvement in the biosafety of the nanoparticles [17,18]. It has also been demonstrated that ND is practically nontoxic to many type of cells, such as human cervical cancer cell line (HeLa), mouse pre-adipocyte (3T3-L1) and osteoprogenitor cell line (489-2) [19], as well as human red blood cells [20] and neuroblastoma cells [3]. Thus, ND could potentially be the part of a drug-delivery system. Herein, we confirmed these observations. In contrast, despite the similar composition of NG and ND, NG is in fact toxic to glioblastoma cells at concentrations ranging from 20 to 100 μg/mL. Interestingly, neither ND nor NG affected the morphology or viability of hepatoma cells. It is an interesting result in terms of using C3A cells as a model of human liver cells in this study. The drug delivery vehicles must be characterized by high biocompatibility and low risk of liver tissue damage. Hepatocytes in the liver, owing to their biotransformation properties, should not be exposed to any toxic xenobiotics. Moreover, we did not observe any significant differences between the performance of both nontransduced and EGFP-expressing U87 and C3A cell lines. Our results support the idea that stable fluorescent cell lines can be employed in cytotoxicity tests as well as their nontransduced counterparts.

We did not observe any significant differences in morphology of U87 or C3A cells after 2 and 24 h of culturing in a medium with NG and ND. The NPs formed aggregates inside or outside the cells, but did not affect their structure. The mechanism of NPs internalization by cells is not completely understood. The main features affecting this process are as follow: NPs size, charge, shape and type of both NPs and cells [21–24]. From the present measurements, it is not possible to conclude whether the NPs entered the cells or were agglomerated on the cell surface. It has been suggested that NPs do not enter the cells [25], however, it was also demonstrated that NPs could be absorbed into the cells either through endocytosis [26,27] or phagocytosis [28,29]. The exemplary mechanism described by Wang et al. in 2011 [30] was based on endocytosis. The authors observed graphene oxide (GO) particles inside human fibroblasts (HDF), mainly located in the cytoplasm, lysosomes, mitochondrion and endoplasm. The amounts of GO inside the fibroblasts gradually increased during the culture period. The authors have suggested following mechanism: GO attachment to the surface of the cells → signal transduction to the nucleus → down-regulation of adhesive proteins’ synthesis → detachment, floating, and shrinking of the cells → GO enter into the cytoplasm (endocytosis) → disturbances in cell energy metabolism, gene transcription and translation → cell death. In turn, Panessa-Warren et al. [31] showed that direct contact between carbon NPs and plasma membrane led to its focal dissolution, allowing small nanoparticles to enter the cytosol and, ultimately, the nucleus. The NPs entered the colon and lung cells on the mechanism independent of the phagocytosis and freely traveled within cytoplasm.

Some authors have reported that carbon NPs could cause the fluorescence quenching, however, in our case, this effect was not so clear. Wang et al. [32] showed that graphene microsheets derived from chemically reduced graphitic oxide (rGO) could effectively decrease the fluorescence intensity in the process that was linearly dependent on the concentration of rGO. Singh et al. [33] compared carbon nanotubes and their capabilities as quencher agents for different fluorophores. Their results showed that the quenching efficiency depends on both the type of NPs and the fluorophores used, and can reach almost 100%. We could not confirm these observations for ND or NG.

The MTT cell viability test demonstrated that NG, at concentrations from 20 to 100 μg/mL, decreased the viability of glioblastoma cells, both nontransduced and EGFP-positive, but did not affect labeled and unlabeled hepatoma cells. There is currently little empirical evidence on cytotoxicity of graphite nanoparticles. Graphite toxicity was not confirmed in rodent lungs after inhalation [34,35], but *in vitro* experiments with some forms of graphite NPs demonstrated differential effects or lack of toxicity depending on various factors: NPs preparation process, size, type of cells, etc. [25,28,30,36,37]. We can speculate that in our experiments observed cytotoxic effect of NG, when compared to ND, may result from the presence of highly reactive dangling carbon bonds on its surface. Interestingly, also here this effect is cell-dependent. To confirm this hypothesis a more detailed chemical and physical analysis of these NPs must be performed. Especially, when biological activity of nanomaterials from different studies is compared, deepened characterization of NPs chemical composition, purity, size distribution, shape and aggregation, surface reactivity, etc. is required. Additionally, since the effects of NPs exposure depend on the target cell type, the understanding of differences between cell membrane structure, function and physiology must be taken into consideration in the assessment of NPs safety.

ND and NG used in our experiments have different zeta potentials. This can suggest that NG, in opposite to ND, is positively charged. Tatur et al. [38] have recently shown that gold NPs functionalized with cationic head groups, in contrast to the NPs functionalized with anionic head groups, penetrated into the hydrophobic moiety of the lipid bilayers and caused membrane disruption at an increased concentration. However, we do not know the real surface charge of our NPs in culture medium. Another factor that may contribute to the NG cytotoxicity in glioblastoma cells is an aggregation of the NPs. NG has higher PDI value than ND and its tendency to aggregate was confirmed by the TEM images.

However, results from experiments that employed graphene to study cytotoxicity are currently available. To some extent, the chemical properties of graphene are similar to those of graphite, owing to the fact that, in both cases, C-atoms are sp^2^ hybridized. Jaworski et al. [39] published their work on the influence of graphene platelets on the morphology, mortality, viability, membrane integrity and type of cell death. Our studies on graphite partially confirmed their results in the case of the MTT test and morphology observations of glioblastoma cells treated with graphene. Both graphene and NG decrease the viability of glioblastoma cells and form agglomerates on the cells surface or inside the cells. On the other hand, the metabolic activity level was not lowered in hepatoma cells after incubation with NG. Lammel et al. [40] tested the cytotoxicity of graphene oxide and carboxyl graphene NPs on hepatoma cells (HepG2). They observed cytotoxic effects such as plasma membrane damage, cell proliferation inhibition and cell death. Nevertheless, the total protein measurement remained unchanged. Another group worked on HepG2 and graphitic nanomaterials (graphene oxide and single-walled carbon nanotubes—SWCNTs), and concluded that graphene had only a moderate effect on the cellular functions when compared to SWCNTs and did not induce apoptosis based on the protein profile [41].

Using ND at concentrations from 20 to 100 μg/mL did not have any negative effect on glioblastoma and hepatoma cells, both nontransduced and EGFP-positive. The cytotoxicity of ND at different concentrations and in different cell types has been discussed in many published articles. Studies of short-term exposure to medium containing ND did not show any significant influence on neuroblastoma, macrophage, keratinocyte [3,42], or hepatoma [43] cells. Some authors suggest that ND after internalization persists nonreactive inside the cell, i.e., in the cytoplasm, and does not harm mitochondria and other organelles [3].

Analysis of the MTT results also revealed an interesting contrast in behavior between nontransduced and EGFP-positive cells. After 2 h of incubation with ND and NG, U87 cells behaved significantly differently to U87-EGFP cells. We observed a small (about 15%), yet significant (*P*<0.05), increase in U87-EGFP cell viability after 2 h of ND exposure at all concentrations. The effect had gone after 24 h. No significant interaction between transduced and nontranduced U87 and C3A cells was observed. We are not able to explain this EGFP-protective phenomenon and we only can hypothesize about some possible interactions between EGFP folding and the NPs. De et al. [44] demonstrated protein refolding through electrostatic interactions after the addition of gold NPs.

The CI represents the status of the cells based on changes in their electrical properties. We analyzed the results obtained for cells cultured for 1 and 2 h after the addition of NPs. The CI in glioblastoma cells decreased after 2 h of incubation with 100 μg/mL NG and after 1 and 2 h of incubation with 50 and 100 μg/mL ND. We did not observe a CI decrease in U87-EGFP cells, except for after 1 h incubation with 50 and 100 μg/mL ND. These results confirm the data obtained from the MTT test, whereby U87-EGFP cells were more viable than nontransduced cells. The opposite situation was observed in hepatoma cells, when the CI increased after incubation with the highest concentration of NG and ND in nontransduced as well as in EGFP-positive cells.

The BrdU incorporation test quantitatively measures a cells proliferative potential. The impact of the NPs on glioblastoma and hepatoma cells was different, but consistent with MTT and RTCA tests results. Notably, the presence of both ND and NG increased the number of hepatoma cells in S-phase. Glioblastoma cells reacted differently to NPs, and with NG at concentrations of 50 and 100 μg/mL, the number of proliferating cells was lowered. Duan et al. [45] performed a series of experiments on human endothelial cells and silica NPs, and found a significant decrease in the S-phase population after NPs treatment. On the other hand, in their previous report, they showed that silica NPs damaged HepG2 cells through ROS production, but the level of cells in S-phase was higher than in the control groups [46].

Based on data from the MTT test, the CI values and the BrdU incorporation test, we can conclude that NG reduced the viability, adherence and proliferation of U87 cells, but did not affect the hepatoma cells. The only observed negative effect of ND was the adherence reduction in glioblastoma cells. Instead, this type of carbon nanomaterial increased viability, adherence and proliferation of hepatoma cells, which supports our hypothesis that ND fits the requirements for the nontoxic drug carriers.

Albumin, the typical protein synthesized by hepatocytes, serves as a popular indicator of changes in hepatoma cell physiology. A reduction in albumin production in C3A cells under stress conditions, i.e., in the presence of the NG and ND at concentrations ranging from 20 to 100 μg/mL, was more prominent in nontransduced cells than in EGFP-positive cells (ELISA test). Bakowicz-Mitura et al. [47] published their study about the influence of diamond powder on human gene expression. The authors found that, despite high biocompatibility and low cytotoxicity, ND had a high molecular activity and could change the expression of genes responsible for cancer. Wierzbicki et al. [5] confirmed that ND and NG can control gene expression. The authors measured mRNA levels of basic Fibroblast Growth Factor (bFGH) and Vascular Endothelial Growth Factor (VEGF) in heart samples following NPs exposure. The levels of bFGH decreased, whereas the levels of VEGF were unchanged. Yuan et al. [48] published an article about the influence of the ZnO NPs on HepG2 cells. This type of NPs caused, in addition to mitochondrial dysfunction, changes in apoptosis-related proteins expression, e.g.: p-Akt, FOXO4, p-ERK, and p-JNK.

## Conclusions

In this study, we confirmed the toxicity of NG to glioblastoma (U87) cells and showed some positive impact of ND and NG on hepatoma (C3A) cells. However, we observed impaired albumin secretion by C3A cells in the presence of both ND and NG. We did not observe any notable differences between the morphology, viability and physiology of nontransduced and EGFP-expressing cells. Moreover, in some experiments, cells expressing the fluorescent marker had an advantage over their nonlabeled counterparts. We observed higher values of CI in EGFP-expressing glioblastoma cells than in nontransduced cells after NG and ND treatment. Additionally, the albumin level after NG and ND treatment in EGFP-expressing hepatoma cells was higher with respect to nontransduced cells.

Analysis of the results of potential cytotoxicity of the carbon-based NPs in U87 and C3A cells demonstrated their differential interactions and advantages, depending on the cell type. However, more research on physicochemical characteristics of these NPs is needed before firm conclusions can be drawn.
